# Non-Polar *Myxococcus fulvus* KYC4048 Metabolites Exert Anti-Proliferative Effects via Inhibition of Wnt/β-Catenin Signaling in MCF-7 Breast Cancer Cells

**DOI:** 10.4014/jmb.2012.12015

**Published:** 2021-03-19

**Authors:** Juha Park, Hee-Jin Yoo, Ah-Ran Yu, Hye Ok Kim, Sang Cheol Park, Young Pyo Jang, Chayul Lee, Wonchae Choe, Sung Soo Kim, Insug Kang, Kyung-Sik Yoon

**Affiliations:** 1 Department of Biochemistry and Molecular Biology, School of Medicine, Kyung Hee University, Seoul 02447, Republic of Korea; 2 Department of Life and Nanopharmaceutical Sciences, Graduate School, Kyung Hee University, Seoul 02447, Republic of Korea; 3 Department of Oriental Pharmaceutical Science, College of Pharmacy, Kyung Hee University, Seoul 02447, Republic of Korea; 4 Lifetogether Co., Ltd., Chuncheon 24232, Republic of Korea

**Keywords:** *Myxococcus fulvus* metabolites, *n*-hexane fraction, MCF-7, Wnt/β-catenin signaling pathway, anti-proliferation

## Abstract

The Wnt/β-catenin signaling pathway is involved in breast cancer and *Myxococcus fulvus* KYC4048 is a myxobacterial strain that can produce a variety of bioactive secondary metabolites. Although a previous study revealed that KYC4048 metabolites exhibit anti-proliferative effects on breast cancer, the biochemical mechanism involved in their effects remains unclear. In the present study, KYC4048 metabolites were separated into polar and non-polar (ethyl acetate and *n*-hexane) fractions via liquid-liquid extraction. The effects of these polar and non-polar KYC4048 metabolites on the viability of breast cancer cells were then determined by MTT assay. Expression levels of Wnt/β-catenin pathway proteins were determined by Western blot analysis. Cell cycle and apoptosis were measured via fluorescence-activated cell sorting (FACS). The results revealed that non-polar KYC4048 metabolites induced cell death of breast cancer cells and decreased expression levels of WNT2B, β-catenin, and Wnt target genes (c-Myc and cyclin D1). Moreover, the *n*-hexane fraction of non-polar KYC4048 metabolites was found most effective in inducing apoptosis, necrosis, and cell cycle arrest, leading us to conclude that it can induce apoptosis of breast cancer cells through the Wnt/β-catenin pathway. These findings provide evidence that the *n*-hexane fraction of non-polar KYC4048 metabolites can be developed as a potential therapeutic agent for breast cancer via inhibition of the Wnt/β-catenin pathway.

## Introduction

Breast cancer is one of the most common cancers among women [1] and its incidence is increasing every year worldwide. In the past few years, breast cancer has constituted nearly 22.9% of female cancers [2, 3]. Regular treatments for this disease include surgery, chemotherapy, radiation therapy, endocrine therapy, targeted therapy, and other supplementary therapies [4]. However, as most of these traditional treatments have side effects that affect the quality of life, [5] new treatments are needed to improve both quality of life and the prognosis of breast cancer patients.

Wnt/β-catenin signaling is essential for various developmental events during tumorigenesis [6]. Mutations in Wnt/β-catenin signaling that contribute to the abnormal regulation of target gene expression of Wnt/β-catenin have been seen in colon and breast cancers [7]. β-catenin in the nucleus regulates gene expression essential for breast stem cell biology during breast development [8]. In the absence of Wnt ligand, β-catenin levels are effectively regulated by complexes containing adenomatous polyposis coli (APC), axin, and glycogen synthase kinase-3 beta (GSK-3β). These complexes can promote the phosphorylation of β-catenin by casein kinase 1 (CK1) and GSK-3β. Phosphorylated β-catenin is multi-ubiquitinated by E3 ubiquitin ligase β-transducin repeat-containing protein (β-TrCP) and degraded by the 26S proteasome [9]. However, in the presence of a Wnt ligand, Wnt and Frizzled receptors can bind to co-receptor low-density lipoprotein-related protein 5/6 (LRP5/6) and inhibit the activity of a destruction complex that degrades β-catenin [8]. In a stable cytoplasm, β-catenin enters the nucleus and binds to the T-cell factor/lymphoid enhancer-binding factor 1 (TCF/LEF) transcription factor and stimulates the expression of target genes such as c-Myc and cyclin D1 that respond immediately to TCF [10, 11]. Persuasive evidence suggests that this pathway is abnormally upregulated during the development of many types of cancer. Thus, disrupting the Wnt/β-catenin signal represents an effective strategy for new drug development in cancer chemotherapy [12, 13].

*Myxococcus fulvus* KYC4048 is a myxobacterial strain [14] that can produce a variety of bioactive secondary metabolites, including well-known antifungal and antibacterial compounds [15]. For example, epothilone derived from myxobacteria is an anti-cancer agent that can induce apoptosis by acting on microtubules and is a microtubule stabilizer such as taxol [16, 17]. In particular, the semisynthetic epothilone derivative ixabepilone was approved by the United States Food and Drug Administration (USFDA) in 2007 as a treatment for metastatic breast cancer [18].

In the previous study, we found by RNA sequencing analysis that various myxobacterial metabolites of KYC4048 are toxic to breast cancer cells involving WNT2B gene [14]. In the current study, we divided KYC4048 metabolites into several fractions through liquid-liquid extraction. We then determined the anti-proliferative effects of these fractions and explored whether the mechanism behind them is involved with the regulation of Wnt/β-catenin signaling. We found that the non-polar fraction of KYC4048 metabolites affected apoptosis by regulating the Wnt/β-catenin signaling pathway in breast cancer cells.

## Materials and Methods

### Antibodies and Reagents

Extra pure grade *n*-hexane and ethyl acetate were purchased from Duksan Pure Chemical (Korea). HPLC-grade formic acid was purchased from Wako (Japan). HPLC-grade acetonitrile was obtained from Fisher Scientific Korea (Korea). Methanol, 3-(4,5-dimethylthiazol-e-yl)-2,5-diphenyl tetrazolium (MTT), propidium iodide (PI) solution, LiCl, crystal violet solution (1%), 37% formaldehyde, and TRI reagent were obtained from Sigma-Aldrich (USA). A RevertAid First Strand cDNA Synthesis Kit and Power SYBR Green PCR Master Mix were purchased from Thermo Fisher Scientific. An Annexin V-FITC/PI Assay Kit and antibodies against cyclin D1 and E2F-1 were purchased from BD Bioscience (USA). Antibodies against WNT2B, c-Myc, Bcl-2, PCNA, and β-actin were purchased from Santa Cruz Biotechnology (USA). Antibodies targeting GSK3α/β, p-GSK3α/β, β-catenin, PARP, CDK4, and HRP-conjugated anti-mouse and anti-rabbit secondary antibodies were obtained from Cell Signaling Technology (USA).

### Myxobacterial Culture and Preparation of Metabolites

For the preparation of metabolites, stock paper containing the myxobacterial KYC4048 strain was spread on CYS agar medium and incubated at 32°C for 7 days as reported previously [14]. Subsequently, cultured myxobacterial cells and 2% Amberlite XAD-16 resin (Sigma) were cultured in CYS liquid medium for 5 days at 32°C with shaking at 180 rpm. The resin and cells were extracted twice each with 50% and 100% acetone (Sigma). Later, acetone was evaporated from the extract using a rotary evaporator. The resulting residue was dissolved in dimethyl sulfoxide (DMSO) at the various concentrations.

### Sample Preparation

Total extract of KYC4048 was fractionated with *n*-hexane and ethyl acetate via liquid-liquid extraction. First, the extract (5 g) was suspended in 250 ml of water. A similar amount of *n*-hexane was added to the separating funnel. The upper layer of *n*-hexane was collected and the same procedure was repeated three times. The collected *n*-hexane fraction was lyophilized to obtain a dried fraction of 92.7 mg (1.9% yield). Then, ethyl acetate was added to the remaining aqueous layer. The ethyl acetate layer amounted to 312.9 mg (6.3% yield) and the weight of the lyophilized aqueous fraction was 4,342.7 mg (86.9% yield). For ultra-performance liquid chromatography (UPLC) analysis, 20 mg of each sample (total extract, *n*-hexane fraction, ethyl acetate fraction, and aqueous fraction) was dissolved in 1 ml of DMSO. All sample solutions were filtered through a 0.2 μm poly-tetrafluoroethylene (PTFE) syringe filter (UK) before injection into the UPLC system.

### Human Cell Culture

MCF-7 and MDA-MB-231 cells were grown in RPMI-1640 medium (HyClone) supplemented with 10% fetal bovine serum (HyClone), 100 U/ml penicillin, and 100 μg/ml streptomycin. MCF-10A cells were grown in Dulbecco’s modified Eagle’s medium (DMEM)/Ham’s F-12 Nutrient Mixture (Gibco) supplemented with 5%horse serum (Gibco), 2 mM L-glutamine, 10 μg/ml insulin, 0.1 μg/ml cholera toxin, 0.5 μg/ml hydrocortisone, and 20 ng/ml recombinant human epidermal growth factor. Cells were cultured at 37°C in a humidified incubator with 5% (v/v) CO_2_.

### MTT Assay

Cell viability was determined based on the conversion of 3-(4,5-dimethylthiazol-2-yl)-2,5-diphenyltertazolium bromide (MTT) to formazan crystals by mitochondrial enzymes. Cells were seeded into 12-well plates at 2 × 10^5^ cells/well in growth medium and cultured for 1 day. These cells were then treated with various concentrations of KYC4048 metabolite fractions. After 24, 48, and 72 h incubation at 37°C, 100 μl MTT solution (5 mg/ml stock) was added to each well. Plates were then incubated at 37°C for 1 h. Subsequently, culture media were carefully removed and DMSO (150 μl) was added to each well to solubilize blue formazan crystals formed by living cells. Absorbance was measured at 540 nm using an ELISA reader (Multiskan EX; Thermo Lab Systems, USA).

### Colony Formation Assay

MCF-7 cells were seeded at a density of 2 × 10^5^ cells/ml in 6-cm culture dishes, cultured for 1 day, and treated with ethyl acetate and *n*-hexane fractions (10 and 20 μg/ml, respectively) of KYC4048 metabolites for 72 h. MCF-7 cells were seeded into 6-well plates (2,000 cells per well) and cultured in complete medium for 14 days. The medium was refreshed every three days. Thereafter, cells were washed with phosphate-buffered saline (PBS), fixed with paraformaldehyde, and stained with 0.5% crystal violet solution. Images of colonies were taken using an Olympus C-5060 digital camera.

### Western Blot Analysis

Cells were seeded at 2 × 10^5^ cells/ml into 6-cm culture dishes, cultured for 1 day, and treated with 100 μg/ml non-polar, ethyl acetate, or *n*-hexane fractions (20 μg/ml, respectively) of KYC4048 metabolites for 24, 48, and 72 h. These cells were then washed with ice-cold PBS and lysed with a buffer containing 50 mM Tris-HCl (pH 7.4), 150 mM NaCl, 1% Triton X-100, 50 mM NaF, 5 mM sodium pyrophosphate, 1 mM EDTA, 1 mM EGTA, 1 mM DTT, 0.1 mM PMSF, and 0.5% protease inhibitor cocktail. The cell lysates were separated by SDS-PAGE and the proteins were transferred to nitrocellulose membranes (Pall Corporation). The membranes were then blocked with a buffer (Tris-buffered saline/Tween-20 containing 5% skim milk) for 1h at room temperature followed by incubation with the indicated primary antibodies overnight at 4°C. The next day, the membranes were labeled with a secondary antibody. Immunoblots were visualized using an enhanced ECL kit (Santa Cruz Biotechnology).

### Real-Time qRT-PCR

Total RNA was extracted using TRI reagent according to the manufacturer’s protocol. After spectrophotometric quantification, 2 μg total RNA in a final volume of 20 μl was converted to cDNAs using a RevertAid First Strand cDNA Synthesis Kit according to the manufacturer’s instructions. qPCR was performed with SYBR Green PCR Master Mix using a 7500 Real-Time PCR Instrument System (Applied Biosystems, USA). All data were normalized against gene expression of GAPDH as an internal control. Gene-specific primers were as follows: (1) c-Myc, forward, 5’-CAGCTGCTTAGACGCTGGATTT-3’, reverse, 5’-ACCGAGTCGTAGTCGAGGT CAT-3’;(2) cyclinD1, forward, 5’-GCATCTACACCGACAACTCCAT-3’, reverse, 5’-GTTTGTTCTC CTCCGCCTCT-3’; (3) GAPDH, forward, 5’-ATGCCCCCATGTTCGTCATG-3’, reverse, 5’-GCAGGAGGCATTGCTGATGA-3 (Bioneer, South Korea).

### Flow Cytometry for DNA Content Analysis and Annexin V-Fluorescein Isothiocyanate (FITC)/Propidium Iodide (PI) Double Staining

To measure apoptosis, MCF-7 cells were seeded at 2 × 10^5^ cells/ml in 6-cm culture dishes, cultured for 1 day, and treated with 5, 10, or 20 μg/ml *n*-hexane fractions of KYC4048 extract for 72 h. Cells were then harvested, washed with ice-cold PBS, fixed at 4°C for 24 h with ice-cold 75% ethanol, and pelleted by centrifugation at 1,500 ×*g* for 5 min at 4°C. Subsequently, cells were washed with PBS, treated for 1 h with 0.5 μg/ml RNase A in PI buffer, and stained for 30 min in the dark with 20 μg/ml PI at 37°C. Cell cycle distribution was then analyzed on the basis of DNA content using Kaluza flow cytometry software (Beckman Coulter, USA). Apoptosis was also quantified by flow cytometry based on annexin V-FITC and PI double staining. Briefly, MCF-7 cells were exposed to *n*-hexane fractions of KYC4048 metabolites, trypsinized, collected by centrifugation, resuspended in annexin V-FITC binding buffer (500 μl), and incubated at room temperature for 30 min in the dark with 1 μg/ml annexin V-FITC and 10 μg/ml propidium iodide. Samples were then analyzed with the Kaluza flow cytometry software.

### UPLC-PDA-ESI-MS Analysis

A Waters Acquity H-class ultra-performance liquid chromatography (UPLC) system (Waters Corp., USA) with a photodiode array (PDA) detector and a JMS-T100TD (AccuTOF) (Jeol Ltd., Japan) mass spectrometer equipped with an electrospray ionization (ESI) source were used for chromatographic and spectrometric (MS) analyses. Chromatographic separation was carried out on an Acquity UPLC BEH C18 Column (130Å, 1.7 μm, 2.1 mm × 50 mm, Waters Corp.) equipped with an Acquity UPLC BEH C18 VanGuard Pre-column (130 Å, 1.7 μm, 2.1 mm × 5 mm). The mobile phase consisted of 0.1% formic acid water (solvent A) and acetonitrile (solvent B). Gradient conditions of the mobile phase were: 0-1 min, 1%; 1-8 min, 1% to 5%; 8-10 min, 5%, 10-20 min, 5% to 15%; 20-35 min, 15% to 100%; 35-40 min, 100%; 40-41 min, 100-1%; and 41-45 min, 1% of solvent B. The flow rate was 0.3 ml/min. The column oven temperature was maintained at 30°C and the detection wavelength was set at 195 nm. The injection volume was 1.0 μl. Conditions of MS analysis in the positive-ion mode were as follows: scan range, *m/z* 50–1000; desolvating chamber temperature, 250°C; orifice 1 temperature, 80°C; orifice 1 voltage, 80 V; orifice 2 voltage, 10 V; ring lens voltage, 5 V; peak voltage, 1,500 V; detector voltage, 1,950 V; nitrogen gas flow rate, 1.0 l/min (nebulizing gas) and 3.0 l/min (desolvating gas).

### Statistical Analysis

All data are presented as mean values ± SD. The statistical significance of data was analyzed by Student’s *t*-test. In all cases, differences between groups were considered statistically significant when *p*-value was less than 0.05.

## Results

### Anti-Proliferative Effects of Non-Polar Fractions of Myxobacterial KYC4048 Metabolites

**Fig. 1:**
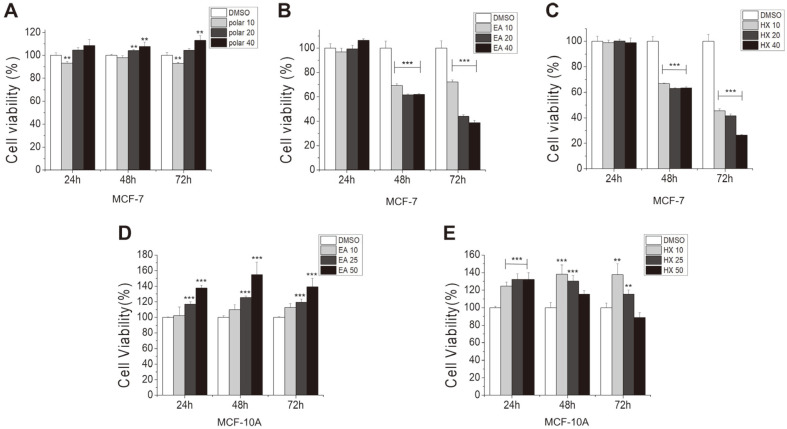
Non-polar fractions of KYC4048 metabolite exert anti-proliferative effects. MCF-7 cells were treated with (**A**) polar, (**B**) ethyl acetate (EA), and (**C**) *n*-hexane (HX) fractions (10, 20 and 40 μg/ml) of KYC4048 metabolites. MTT assay was then performed at 24, 48, and 72 h after treatment. MCF-10A cells exposed to (**D**) EA and (**E**) HX fractions (10, 25, and 50 μg/ml) of non-polar KYC4048 metabolites for the indicated time. Cell viability was then measured by MTT assay. Controls were treated with the corresponding volume of DMSO. Data represent the mean ± SD of at least three experiments. **p < 0.01; ***p < 0.001 compared to the control treated with DMSO.

KYC4048 metabolites were divided into polar and non-polar fractions. The non-polar fractions were divided into ethyl acetate (EA) and *n*-hexane fractions (HX) via liquid-liquid extraction. To determine the anti-proliferative effects of these fractions in MCF-7 breast cancer cells, cell viability was measured by MTT assay. The results showed that the viability of MCF-7 cells exposed to polar fractions (10, 20, and 40 μg/ml) was not decreased (Fig. 1A). However, the viability of MCF-7 cells exposed to EA fractions at 10, 20, and 40 μg/ml were decreased to 72.3 ± 1.5%, 43.9 ± 1.4%, and 39 ± 1.6 %, respectively (Fig. 1B). Similarly, the viability of MCF-7 cells exposed to the HX fractions at 10, 20, and 40 μg/ml were decreased to 45.7 ± 1.4%, 41.4 ± 1.5%, and 26.5 ± 0.6 %, respectively, at 72 h after treatment (Fig. 1C). At similar concentrations, the HX fractions were more potent than the EA fractions in decreasing the viability of MCF-7 cells. To evaluate the toxicity of the EA and HX fractions to immortalized cells derived from other tissues of the breast (MCF-10A), cell viability was measured by MTT assay. Our data showed that MCF-10A cells treated with EA and HX fractions showed more growth than control cells at 72 h after treatment (Figs. 1D and 1E). These findings demonstrate that the non-polar fractions of KYC4048 metabolites exerted anti-proliferative effects on MCF-7 breast cancer cells selectively.

### Non-Polar Fractions of KYC4048 Metabolites Inhibit Colony Formation

**Fig. 2:**
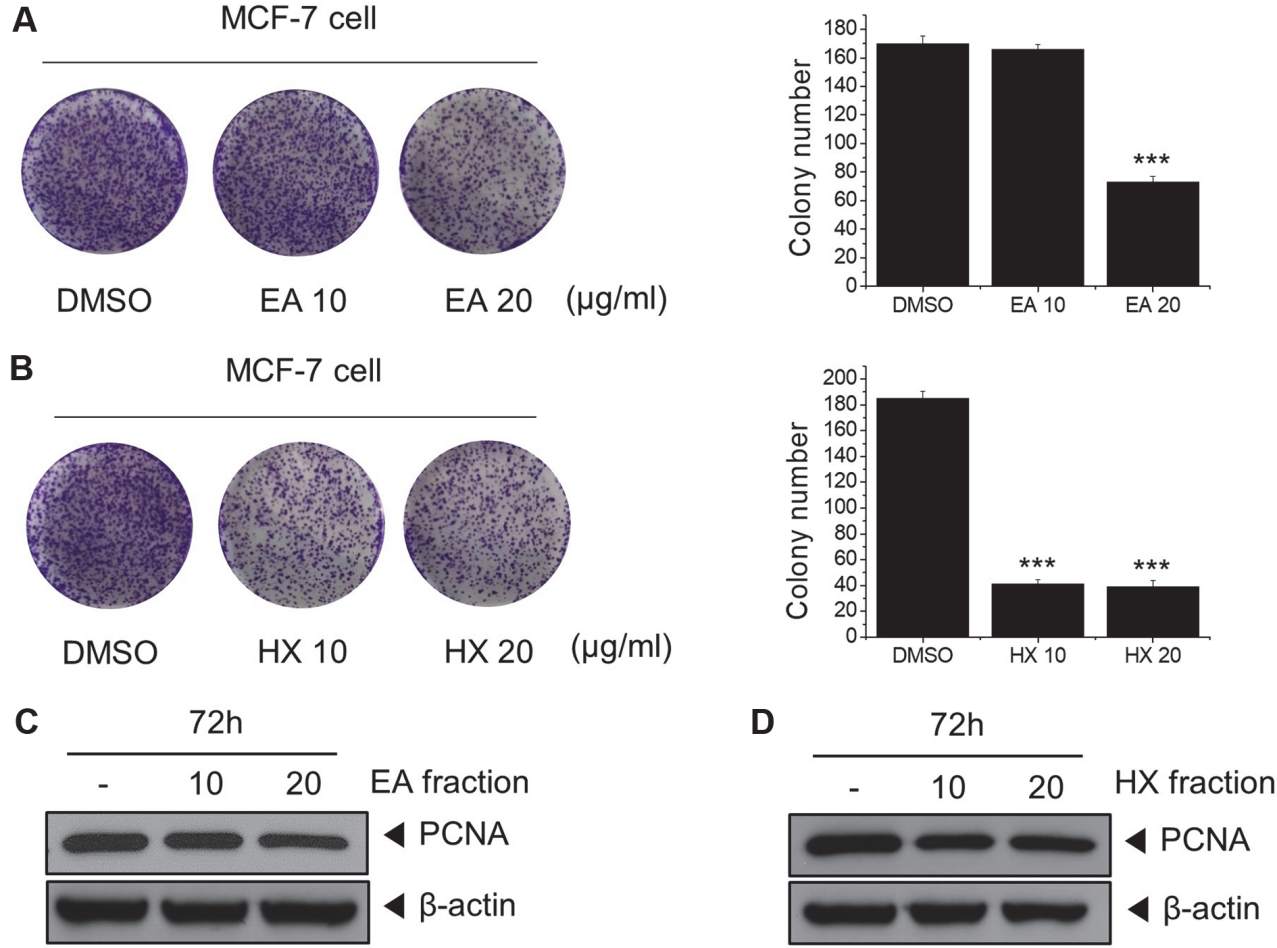
Effects of non-polar fractions of KYC4048 metabolites on colony formation of MCF-7 cells. MCF-7 cells were exposed to different concentrations of (**A**) EA fraction and (**B**) HX fraction (10 and 20 μg/ml) for 72 h. After seeding (2,000 cells/well), cells were observed for two weeks. MCF-7 cells were treated with (**C**) EA fraction and (**D**) HX fraction (10 and 20 μg/ml) for 72 h. Antibody specific to PCNA was used for Western blot analysis. β-actin was used as a loading control.

To explore whether non-polar fractions (EA and HX) could inhibit colony formation of MCF-7 cells, we performed colony formation and Western blot assay. MCF-7 cells were exposed to different concentrations (10, 20 μg/ml) of EA and HX fractions for 72 h. Cells were then observed for two weeks. Results showed that the colony formation ability was decreased by EA and HX fractions (Figs. 2A and 2B). Expression levels of the proliferation protein marker PCNA were decreased in MCF-7 cells treated with EA or HX fractions (10, 20 μg/ml) for 72h (Figs. 2C and 2D). These results suggest that non-polar fractions of KYC4048 metabolites can inhibit the proliferation of MCF-7 cells with a long-term effect.

### Non-Polar Fractions of KYC4048 Metabolites Inhibit Wnt/β-Catenin Signaling Pathway

**Fig. 3:**
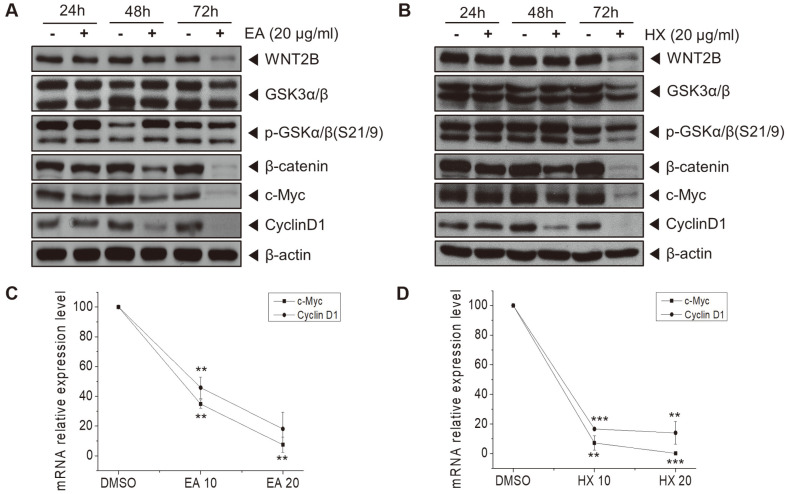
Non-polar fractions of KYC4048 metabolites inhibit Wnt/β-catenin signaling pathway. MCF-7 cells were treated with (**A**) EA fraction (20 μg/ml) and (**B**) HX fraction (20 μg/ml) of KYC4048 metabolites for the indicated time. WNT2B, GSK-3α/β, p-GSK-3α/β, β-catenin, c-Myc, and cyclin D1 protein levels were detected by Western blot. β-actin was used for a loading control. MCF-7 cells were treated with 10 or 20 μg/ml (**C**) EA fraction and (**D**) HX fraction, respectively, for 72 h. Total RNA was extracted from cells and converted to cDNA. mRNA expression levels of c-Myc and cyclin D1 were then determined by quantitative PCR analysis. Data are presented as mean ± SD of at least three experiments. **p < 0.01; ***p < 0.001 compared to the control treated with DMSO.

To examine whether non-polar fractions of KYC4048 metabolites could regulate the Wnt/β-catenin signaling pathway, we performed Western blot analysis and real-time qRT-PCR. MCF-7 cells were treated with EA and HX fractions at 20 μg/ml. The results showed that protein expression levels of WNT2B, p-GSK-3β, β-catenin, and target genes c-Myc and cyclin D1 were decreased by both fractions at 72 h after treatment (Figs. 3A and 3B). After MCF-7 cells were treated with EA and HX fractions at 10 and 20 μg/ml for 72 h, mRNA expression levels of the c-Myc and cyclin D1 were also decreased by both fractions (Figs. 3C and 3D). These results suggest that these non-polar fractions could inhibit the Wnt/β-catenin signaling pathway. In addition, the HX fraction has more potential than the EA fraction in inhibiting the Wnt/β-catenin signaling pathway.

### *n*-Hexane Fraction of KYC4048 Metabolites Induces Cell Cycle Arrest

**Fig. 4:**
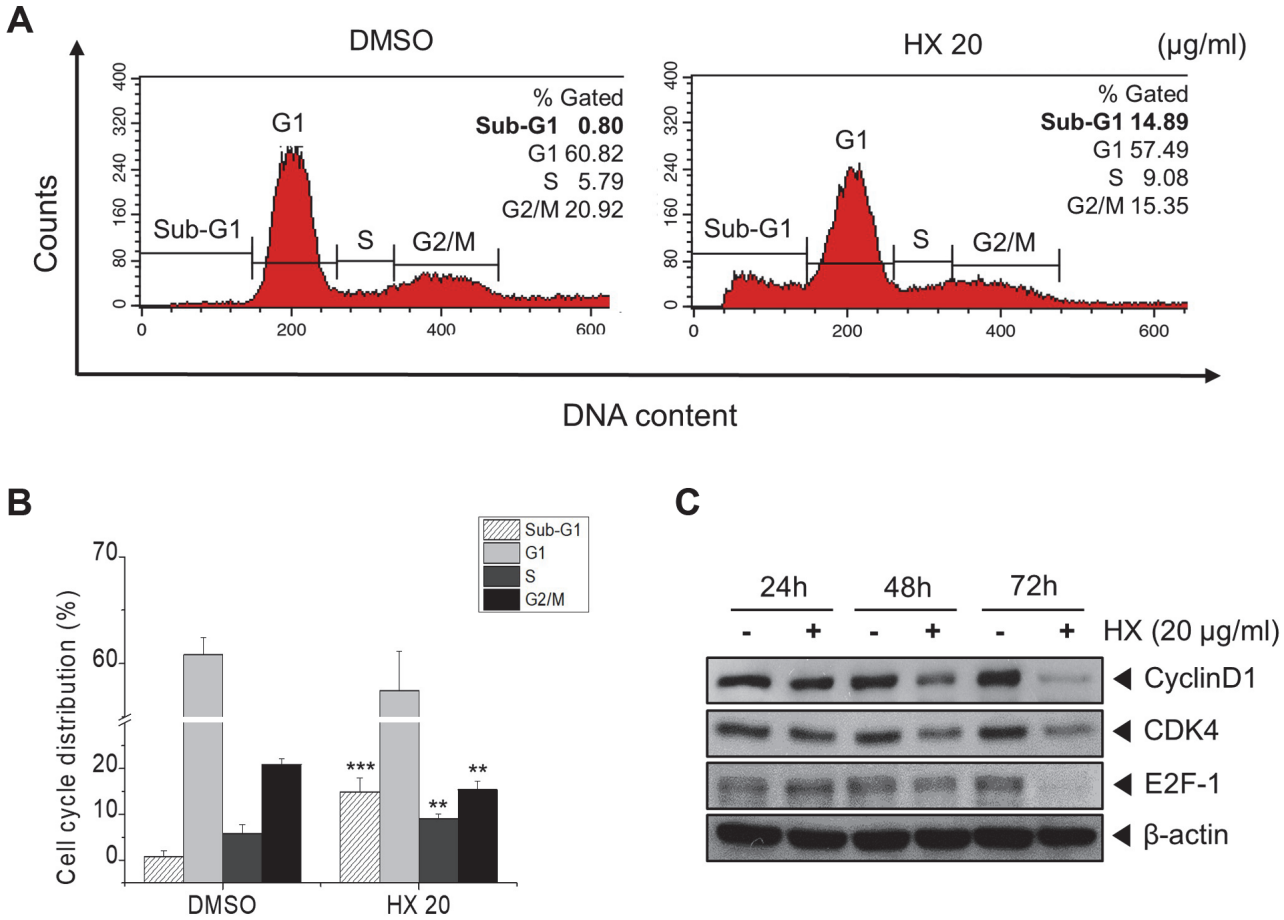
Treatment with *n*-hexane fraction of KYC4048 metabolites induces cell cycle arrest. MCF-7 cells were treated with (**A**) DMSO and HX fractions (20 μg/ml) and then stained with PI at 72 h. DNA content was measured by flow cytometry. (**B**) Proportions of MCF-7 cells in sub-G1, G1, S, and G2/M phases. (**C**) Expression levels of cyclin D1, CDK4, and E2F-1 detected by Western blot analysis after treatment with 20 μg/ml HX fraction for the indicated time. β-actin was used as a loading control. Data are presented as mean ± SD of at least three experiments. **p < 0.01; ***p < 0.001 compared to the control treated with DMSO.

To determine whether the HX fraction induced cell cycle arrest in MCF-7 cells, we investigated cell cycle distribution via flow cytometry. As shown in Figs. 4A and 4B, cell counts was increased in the sub-G1 phase (14.89%) by the HX fraction compared to that in the control at 72 h. Subsequently, to determine cell cycle arrest in MCF-7 cells, the protein levels of cell cycle markers were measured by Western blot analysis. The protein levels of cyclin D1, CDK4, and E2F-1 as cell cycle markers were decreased by treatment with the HX fraction at 72 h (Fig. 4C). These results suggest that the HX fraction of KYC4048 metabolites could induce sub-G1 cell cycle arrest in MCF-7 cells.

### *n*-Hexane Fraction of KYC4048 Metabolites Induces Cell Death

**Fig. 5:**
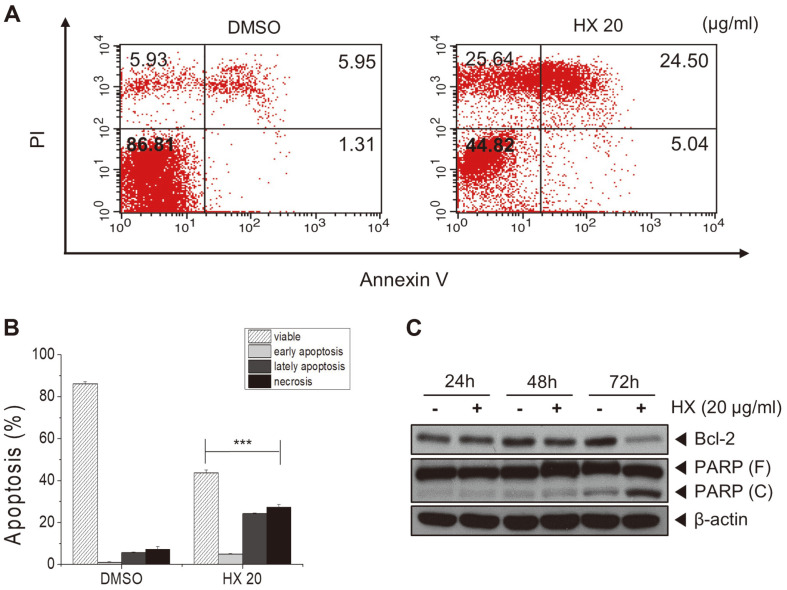
Treatment with *n*-hexane fraction of KYC4048 metabolites induces cell death. (**A**) MCF-7 cells were treated with 20 μg/ml HX fraction of non-polar KYC4048 metabolites, stained with annexin V-FITC/PI, and then analyzed by flow cytometry at 72 h. (**B**) Graphical representation of apoptotic and necrotic percentages of MCF-7 cells. (**C**) MCF-7 cells were treated with 20 μg/ml HX fraction. Expression levels of anti-apoptotic proteins Bcl-2 and PARP were determined by Western blot analysis at 24, 48, and 72 h. β-actin was used as a loading control. Data are presented as mean ± SD of at least three experiments. ***p < 0.001 compared to the control treated with DMSO.

To explore whether apoptosis and necrosis of MCF-7 cells could be induced by treatment with *n*-hexane fractions, annexin V-FITC/PI double staining assay was performed. The results showed that percentage of annexin-/pi+ (necrosis) and annexin+/pi+ (late apoptosis) is increased to 25.64% and 24.50% compared to those in the control (5.93, 5.95%) after treatment with the HX fraction at 72 h (Figs. 5A and 5B). The protein expression levels of cleaved-PARP and Bcl-2 were then measured by Western blot anslysis. The results showed that the expression level of anti-apoptotic protein Bcl-2 is decreased whereas those of cleaved-PARP is increased at 72 h (Fig. 5C). These results suggest that the HX fraction of KYC4048 metabolites can induce death of MCF-7 cells.

### Inhibition of Wnt/β-Catenin Signaling Pathway via GSK-3β-Dependent Mechanism by the *n*-Hexane Fraction of KYC4048 Metabolites

**Fig. 6:**
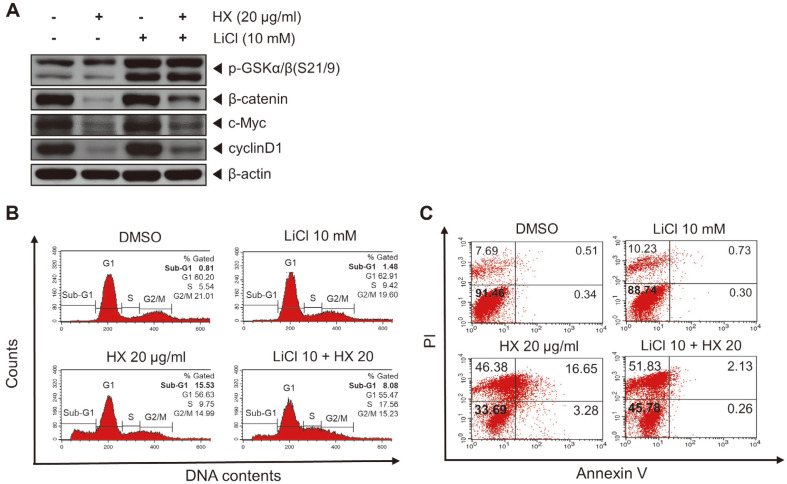
*n*-Hexane fraction of KYC4048 metabolite suppresses Wnt/β-catenin signaling pathway via GSK3β- dependent mechanism. (**A**) MCF-7 cells were co-treated with LiCl (10 mM) and *n*-hexane fraction (20 μg/ml) for 72 h. Protein levels of p-GSK-3α/β, β-catenin, c-Myc, cyclin D1, and β-actin were detected by Western blot analysis. (**B**) MCF-7 cells were co-treated with 10 mM LiCl and 20 μg/ml *n*-hexane fraction and stained with PI at 72 h after treatment. DNA content was analyzed by flow cytometry. (**C**) MCF-7 cells were treated with 10 mM LiCl and 20 μg/ml *n*-hexane fraction of non-polar KYC4048 metabolites and stained with annexin V-FITC/PI followed by flow cytometry at 72 h.

To investigate whether the HX fraction of KYC4048 metabolites could inhibit Wnt/β-catenin signaling pathway signaling through the GSK-3β-dependent mechanism, LiCl as a GSK-3β inhibitor was used to treat cells followed by Western blot analysis and flow cytometry analyses. The results showed that the effects of the HX fraction on p-GSK-3β, β-catenin, c-Myc, and cyclin D1 are abolished by LiCl (Fig. 6A). In addition, the sub-G1 phase, which was increased to 15.53% by the HX fraction, was induced up to only 8.08% by LiCl co-treatment. Thus, its effect was blocked by LiCl (Fig. 6B). Morever, the proportion of non-apoptotic cells, which was reduced to 33.69% by the HX fraction, was increased to 45.75% by co-treatment with LiCl (Fig. 6C). These data suggest that the effect of the HX fraction is mediated by GSK-3β in the Wnt/β-catenin pathway.

### Identification of Compounds in the *n*-Hexane Fraction of KYC4048 Metabolites

**Fig. 7:**
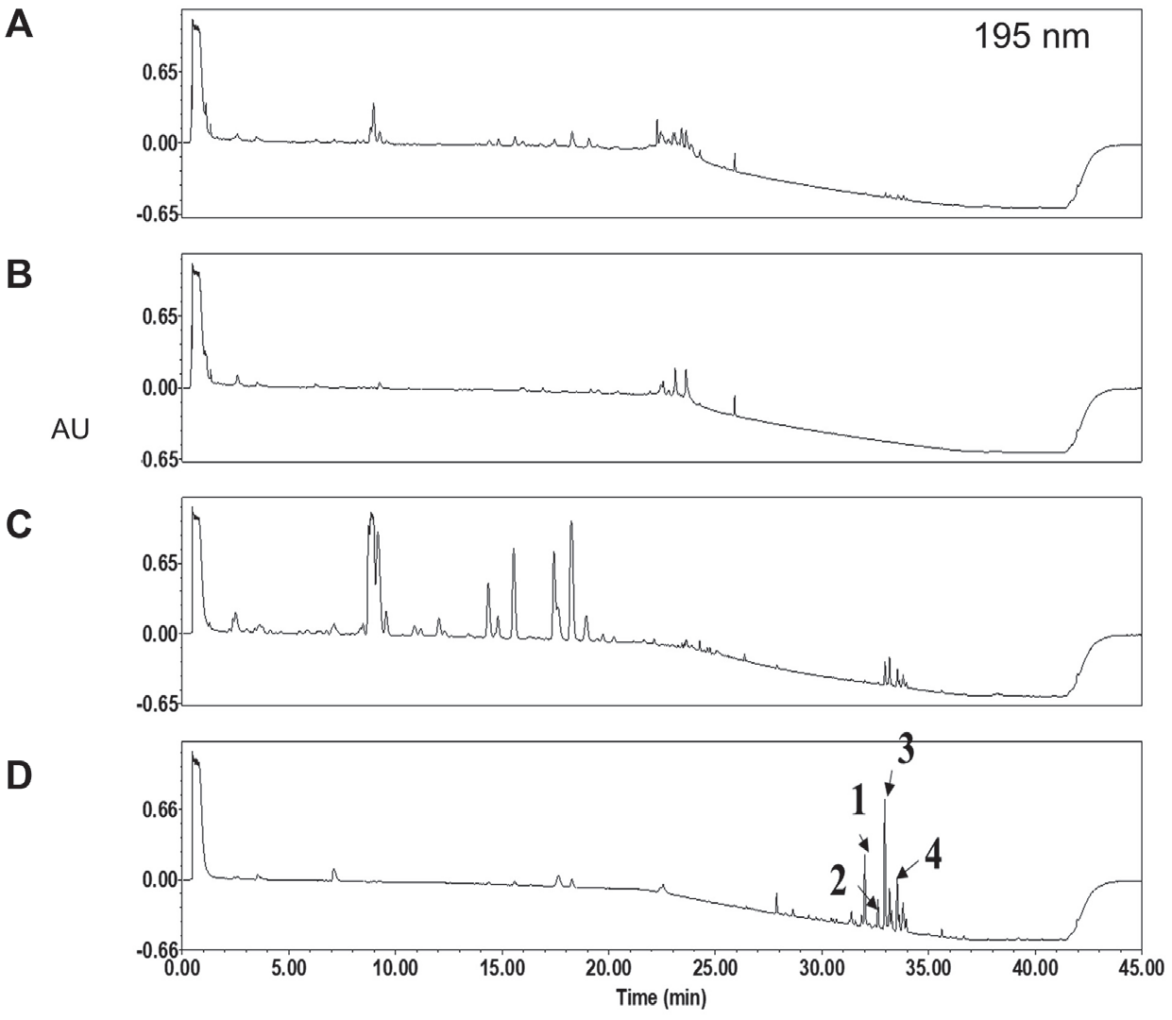
*n*-Hexane of KYC4048 metabolites shows activity peaks. UPLC chromatograms of KYC4048 metabolites, including (**A**) total extract, (**B**) aqueous fraction, (**C**) ethyl acetate fraction, and (**D**) *n*-hexane fraction, are shown. These chromatograms were monitored at 195 nm.

We performed UPLC to investigate the active substances in each KYC4048 metabolite fraction. Optimized UPLC chromatograms of (A) total extract, (B) aqueous (polar) fraction, (C) ethyl acetate fraction, and (D) *n*-hexane fraction are shown in Fig. 7A. Although the aqueous fraction contained most of the total extract in terms of dry weight (86.9%), a few peaks of UV-absorbing organic compounds were detected. Both the ethyl acetate and *n*-hexane fractions showed several peaks of UV-absorbing compounds, indicating that more diverse metabolites are contained in these fractions. A UPLC-ESI-MS study was performed for the *n*-hexane fraction which showed the most potent activity. Four major peaks were selected. Retention time (Rt), mass number of precursor ion, and possible molecular formulas of these peaks are listed in Table 1.

## Discussion

Myxobacteria can secrete a variety of active secondary metabolites to defend themselves from other microorganisms or eukaryotes [19, 20]. Previous studies have shown that metabolites secreted by the myxobacterial strain KYC4048 possess anti-proliferative effects on breast cancer cells. Therefore, in the present study, to investigate compounds responsible for the anti-proliferative effect on MCF-7 cells, we separated KYC4048 metabolites into several fractions by difference in polarity. Our results showed that: 1) non-polar fractions of KYC4048 metabolites exerted anti-proliferative effects on MCF-7 cells, with the *n*-hexane fraction showing the most potent activity; 2) the *n*-hexane fraction inhibited the Wnt/β-catenin signaling pathway; and 3) the *n*-hexane fraction induced cell cycle arrest, apoptosis, and necrosis. We also measured cell viability afer treating MDA-MB-231 triple-negative breast cancer cells with polar, EA, and HX fractions. We found that cell viability are decreased by EA and HX fractions dose-dependently after treatment for 72 h, although these fractions are less effective on MDA-MB-231 cells than on MCF-7 cells (data not shown).

Abnormal activation of the Wnt/β-catenin signaling pathway can advance breast cancer [21]. Various studies have reported that specific natural compounds can induce apoptosis of breast cancer cells by inhibiting the Wnt/β-catenin signaling pathway. Prodigiosin, a red pigment produced by bacteria, has been shown to exhibit anti-cancer effects by inhibiting the Wnt/β-catenin signaling pathway in breast cancer both in vitro and in vivo [22]. A derivative of goniothalamin known as 5-acetyl goniothalamin (5GTN) can suppress the proliferation of breast cancer cells via inhibition of the Wnt/β-catenin signaling pathway [23]. Diallyl trisulfide, a garlic oil-soluble sulfur ingredient, has been reported to inhibit breast cancer stem cells via inhibition of GSK-3β-mediated Wnt/β-catenin pathway [24]. Therefore, inhibiting the Wnt/β-catenin signaling pathway is a promising strategy in breast cancer chemotherapy. In the present study, we found that levels of WNT2B, p-GSK-3β, β-catenin, and target genes c-Myc and cyclin D1 were decreased in MCF-7 cells treated with EA and HX fractions of KYC4048 metabolites. In the Wnt/β-catenin signaling pathway, glycogen synthase kinase-3β (GSK-3β) can catalyze β-catenin phosphorylation at Ser33/37 residues, resulting in ubiquitin-dependent proteasomal degradation [25]. We found that the suppression of Wnt/β-catenin signaling pathway-related protein and the induction of cell cycle arrest and apoptosis in MCF-7 cells by the HX fraction are abolished by LiCl. These data suggest that GSK-3β plays a vital role in suppressing the Wnt/β-catenin signaling pathway by the HX fraction. Nevertheless, the previous studies showed that the expression level of β-catenin protein is not decreased by myxobacteria KYC4048 metabolites. It has been explained that Wnt/β-catenin signaling is involved in increasing the expression of p-β-catenin by KYC4048 metabolites. However, we used a non-polar fraction of KYC4048 metabolites. There may be substances that stimulate Wnt/β-catenin signaling even more. Unlike previous papers, the expression level of β-catenin was decreased significantly after treatment with KYC4048 metabolites in the present study.

Intrinsic apoptosis is mediated by mitochondria. It is mainly regulated by the Bcl-2 protein family. Under normal conditions, Bcl-2 can inhibit the pore formation of Bax and Bak. When BH3-only proteins are increased and activated by signals that stimulate intrinsic apoptosis, mitochondrial outer membrane permeabilization (MOMP) formation will occur via inhibition of Bcl-2 or activation of Bax and Bak. Apoptosome formation occurs when cytochrome c is leaked into the cytoplasm through MOMP. The apoptosome can activate caspase-9, along with executor caspases such as caspase-3, caspase-7, and cleaved-PARP, resulting in activation of apoptosis [26]. In the present study, we found that treatment with *n*-hexane fraction induces death of MCF-7 cells via regulation of Bcl-2 and PARP. However, when we investigated, caspases and Bax (apoptosis-related molecules), the cleaved-form of caspases and Bax did not increase (data not shown). Therefore, further studies are needed to understand the mechanisms related to apoptosis. In addition, fractions of KYC4048 metabolites contain many compounds. We focused on the efficacy of these fractions rather than the exact molecular pathway. Our results confirmed that necrosis is induced by the *n*-hexane fraction through FACS, although the mechanism for necrosis and apoptosis is unclear. Further research is needed. Cepharanthine, a natural alkaloid extracted from *Stephania cepharantha* Hayata, can induce cell cycle arrest in the G0/G1 phase by inhibiting cyclin D/CDK complexes in breast cancer cells [4]. In this study, we confirmed that cell cycle arrest is induced in the Sub-G1 phase and that levels of cyclin D1, CDK4, and E2F-1 are reduced by the *n*-hexane fraction.

Various groups have used the UPLC system to analyze new compounds. In these studies, few peaks of UV-absorbing organic compounds were detected in the aqueous fraction, indicating the presence of polysaccharides, proteins, and other polar primary metabolites. In contrast, four major peaks were identified in the *n*-hexane fraction by UPLC in the present study. Based on the mass number of precursor ions and possible molecular formulae, these peaks were not characteristics of epothilone derivatives. The chemical identities of these compounds need to be elucidated via further chromatographic and spectroscopic studies. The in vivo effectiveness of the *n*-hexane fraction also needs to be determined in the future.

In conclusion, our data showed that non-polar fractions of KYC4048 metabolites exert anti-proliferative effects on MCF-7 cells, with the *n*-hexane fraction showing the most potent activity. We also found that the *n*-hexane fraction could inhibit the Wnt/β-catenin signaling via GSK-3β in breast cancer cells. Further, using UPLC, we identified four major peaks in the *n*-hexane fraction. Taken together, our results demonstrate that the *n*-hexane fraction of KYC4048 metabolites can exert a potent anti-proliferative effect, and therefore represent a potential therapeutic agent for treating breast cancer.

## Figures and Tables

**Table 1:** Retention time (Rt), precursor ion, and possible molecular formulas of peaks.

Peak	Rt (min)	Precursor ion *(m/z)*	Suggested Molecular Formulae
1	32.00	398.23098 [M+H]^+^	C_12_H_32_NO_4_ C_22_H_30_N_4_O_3_ C_25_H_28_N_5_ C_12_H_34_O_7_ C_20_H_28_N_7_O_2_
2	32.62	297.28898 [M+H]^+^	C_16_H_35_N_5_ C_18_H_37_N_2_O
3	32.93	282.27838 [M+H]^+^	C_16_H_34_N_4_ C_18_H_36_NO
4	33.52	358.30975 [M+H]^+^	C_21_H_42_O_4_
